# Transcorneal freezing and topical Rho-kinase inhibitor treatment in Fuchs endothelial corneal dystrophy

**DOI:** 10.1038/s41433-021-01520-2

**Published:** 2021-04-12

**Authors:** Johannes Menzel-Severing, Stefan Schrader, Ursula Schlötzer-Schrehardt, Gerd Geerling

**Affiliations:** 1 Department of Ophthalmology, Heinrich Heine University of Düsseldorf, Düsseldorf, Germany; 2 Department of Ophthalmology, Carl von Ossietzky University Oldenburg, Oldenburg, Germany; 3 Department of Ophthalmology, University of Erlangen-Nuremberg, Erlangen, Germany

**Keywords:** Surgery, Drug therapy

## To the Editor:

Posterior lamellar keratoplasty has become the standard of care for surgical treatment of corneal endothelial cell (CEC) disease such as Fuchs endothelial corneal dystrophy (FECD). Despite excellent outcomes, potential complications (e.g., graft detachment or immune rejection) have prompted research efforts to identify alternatives. It has been suggested that Rho-associated protein kinase (ROCK) inhibitors may enable CEC regeneration following removal of central CECs by stimulating proliferation and migration in remaining peripheral cells [1, 2]. Indeed, data from recent clinical studies indicate that removal of Descemet’s membrane (DM) without endothelial keratoplasty can yield sustained recovery of vision when aided by ripasudil (ROCK inhibitor) eye drops [3]. However, it has been observed that endothelial cell proliferation and migration to cover a denuded posterior corneal surface is reduced when DM has been removed [4]. This would speak in favor of removing only the diseased CECs while leaving DM intact. On the other hand, it has been argued that the presence of guttae may also impair CEC healing, as guttae may act as a physical barrier to centripetal migration [5]. Currently, there is no conclusive evidence as to which surgical approach is best combined with ROCK inhibitor treatment to enhance CEC regeneration.

## Methods

We report our findings from three patients who were treated for moderate FECD using the ERBOKRYO^®^ AE cryosurgical system (Erbe Elektromedizin, Tübingen, Germany) at –60 °C applied for 15 s to the central 3 mm of the cornea to remove CECs. This was followed by ripasudil eye drops (Glanatec^®^ ophthalmic solution 0.4%, Kowa Company, Ltd., Nagoya, Japan) administered six times daily for 1 week. Corrected distance visual acuity (CDVA) was obtained using Snellen charts and converted to logMAR.

## Results

Patient 1 (female, age 48) presented with CDVA of 0.3 (logMAR). This increased to 0.1 at 1 month after cryotherapy/ripasudil treatment. Two years after treatment, CDVA dropped to 0.2. Descemet membrane endothelial keratoplasty (DMEK) was performed 3 ½ years after treatment, CDVA being 0.5. Two months after DMEK, CDVA increased to 0.0.

Patient 2 (female, age 62) also presented with CDVA of 0.3. At 1 month after treatment, CDVA remained at 0.3. DMEK was performed 14 months later, CDVA at that point being 0.5. Three months after DMEK, CDVA increased to 0.1.

Patient 3 (male, age 64) presented with CDVA of 1.0. At 6 months after treatment, CDVA had improved to 0.2. At 18 months after treatment, CDVA dropped by one line due to herpes simplex keratitis. This prompted penetrating keratoplasty, which was performed 2 ¾ years after treatment. CDVA at that point was 0.8.

**Fig. 1:**
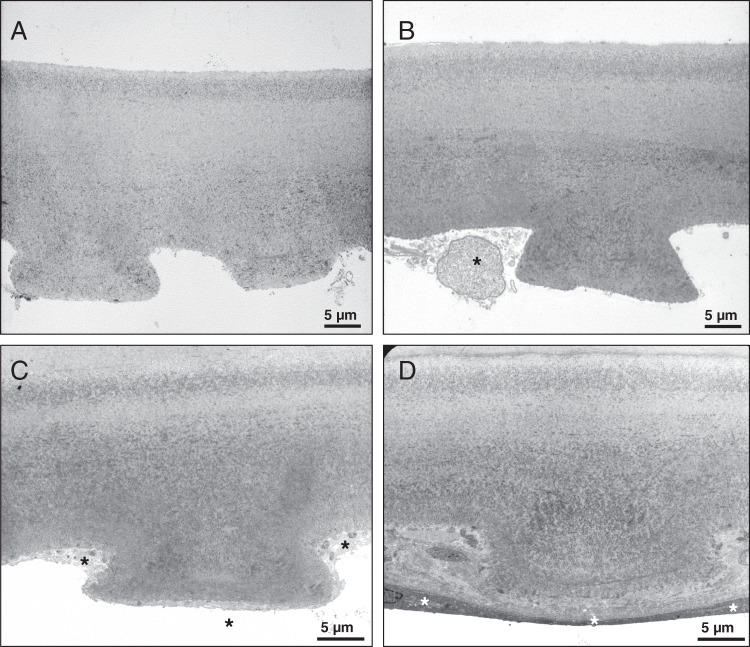
Transmission electron microscopy images of corneal tissue specimens. Descemet’s membrane of Patient 2 shows typical ultrastructural features of cornea guttata (**A**). No overt differences can be observed when comparing with the morphology of Descemet’s membrane removed from the fellow eye (**B**). Descemet’s membrane of Patient 3 also shows typical guttae. No differences can be discerned between the treated central (**C**) and the untreated peripheral cornea (**D**). Asterisks show corneal endothelial cells.

Transmission electron microscopy studies were performed on corneal tissue specimens obtained during surgery from Patients 2 and 3 (Fig. 1). DM ultrastructure in both cases shows alterations typical of FECD but no sequelae of transcorneal freezing and/or ripasudil treatment.

## Discussion

Our observations suggest that transcorneal freezing does not affect the ultrastructural composition of DM in FECD. The persistence of guttae may explain why this procedure yields improvement in CDVA only transiently, despite the application of ripasudil. However, the intervention does not preclude subsequent DMEK surgery with excellent visual outcomes. It may therefore provide a viable option in patients who benefit from postponing intraocular surgery. However, additional studies are required to determine the mean duration of improved visual acuity. Also, it remains to be determined whether patients with endothelial disease not characterized by corneal guttae may have a more sustained effect.
